# Determining the sweet detection threshold of COVID-19 patients during infection and recovery periods

**DOI:** 10.1371/journal.pone.0309342

**Published:** 2024-08-29

**Authors:** Woroud Alsanei, Esraa Alhussin, Zuhair S. Natto, Marwah Afeef, Tugba Aktar

**Affiliations:** 1 Department of Food and Nutrition, Faculty of Human Sciences and Design, King Abdulaziz University, Jeddah, Saudi Arabia; 2 Department of Nursing Education, King Fahad Hospital Jeddah, Jeddah, Saudi Arabia; 3 Department of Dental Public Health, Faculty of Dentistry, King Abdulaziz University, Jeddah, Saudi Arabia; 4 Department of Research and Studies, Al-Thagher Hospital, Jeddah, Saudi Arabia; 5 Department of Food Engineering, Faculty of Engineering, Alanya Alaaddin Keykubat University, Antalya, Turkey; Taichung Veterans General Hospital, TAIWAN

## Abstract

**Background:**

The loss of taste and smell is a common symptom of COVID-19, affecting individuals’ quality of life and nutritional status. Detecting sweet thresholds during infection and recovery periods can assist in implementing dietary modifications and nutritional strategies for these patients.

**Objective:**

To investigate the changes and differences in sweet detection thresholds of confirmed COVID-19 patients on Day 1, Day 10, and Day 14 of the infection and recovery periods.

**Methods:**

The demographic factors such as gender, smoking status, BMI, and age group were abstracted on Excel sheet from the medical health records for confirmed COVID-19 patients, who were admitted to King Fahad General Hospital in Jeddah, Saudi Arabia, a COVID-19 care facility, from September 2021 to July 2022. Sweet detection thresholds were determined using a pair-wise comparison procedure and sugar solutions with varying concentrations, arranged in ascending order and presented to participants until the lowest detected concentration was noted after three consecutive positive detections, with the median just noticeable difference (JND) value calculated as the population average threshold. Sensory tests were conducted on COVID-19 patients during their infection and recovery periods to evaluate their taste sensation thresholds. The demographic factors of gender, smoking status, BMI, and age group were considered in the analysis.

**Results:**

A total of 37 patients who met the inclusion criteria of the study were enrolled as participants. Significant variances in sweet detection thresholds were observed among the COVID-19 patients, with consistent decreases over the three testing days, indicating increasing sucrose sensitivity. Infected men showed significant returns to sweet detection thresholds on Day 14 compared to women, while infected smokers exhibited greater recoveries than non-smokers. Overweight patients had consistently elevated thresholds and recovery rates that were comparable to those of normal-weight patients by Day 14, while younger patients had lower thresholds than their older counterparts. On Day 14, the thresholds had significantly recovered to a level comparable to that of healthy individuals (approximately 0.23%).

**Conclusion:**

These findings suggest that sweet detection thresholds can be used as a marker for assessing the progression and recovery of COVID-19 patients. These findings highlight the importance of recognizing and managing alterations in sweet detection thresholds promptly in COVID-19 patients, as this could positively impact dietary management, nutritional recommendations, and interventions during infection and recovery periods.

## Introduction

Sweetness is a main mammalian taste type that is used to sense and perceive preferred foods. Previous research has suggested that sweetness is a favorite of the taste types and one of the earliest sensed [1]. Sweet taste is created by sweetener molecules, which mainly include carbohydrates such as glucose, fructose, and sucrose as well as amino acids such as glycine [2]. Sweetness is a primitive sense with straightforward sensory mechanisms that has been used as a primary signal to assess sensory mechanisms in humans, including those involving discriminative, descriptive, or hedonic tests. Several factors influence the perception of sweetness, such as sweetener concentration, serving temperature [3–5], saliva and food pH [6, 7], consumer age [1, 8], consumer genetics [9, 10], food ingredients and physicochemical media [9, 11, 12], and consumer health condition such as chronic diseases or infections that affect the taste buds [13–19].

The COVID-19 pandemic was removed as a Public Health Emergency of International Concern (PHEIC) in the World Health Organization (WHO) Director-General’s report of the 15^th^ meeting of the IHR Emergency Committee on May 5^th^, 2023, due to decreasing numbers of deaths and hospitalizations and high levels of immunity in the population [20]. The WHO statistics reveal 767,726,861 confirmed COVID-19 cases and 6,948,764 deaths, with the highest number of cases in Europe, followed by the Western Pacific, the Americas, South-East Asia, the Eastern Mediterranean, and Africa [21]. Apart from the severe consequences, a significant proportion of patients reported the loss of smell or taste [22–28] and official reports have confirmed that temporary losses or impairments in these senses are symptoms of COVID-19 [25]. COVID-19 recovery times are uncertain, with variations between 10 to 14 days and several months of the median time to chronic loss of smell or taste [29–31].

Loss or impairment of taste are common symptoms of COVID-19 infection; however, little is known regarding the differences and correlations in sweet detection thresholds across different patient groups and during the infection and recovery periods. This study aimed to evaluate the detection thresholds of sweet taste loss among confirmed COVID-19 patients using a detection threshold based on increasing sucrose concentrations. Sensory sweet tests of diagnosed patients were conducted on Day 1, Day 10, and Day 14 of the infection and recovery to investigate any changes in tasting ability, with consideration of the influencing factors of gender, smoking status, BMI, and age group, which were anticipated to result in variabilities in the detection thresholds.

## Materials and methods

### Study population

The study design involved a case series of 37 confirmed COVID-19 patients who had received their second COVID vaccine. Prior to participating in a sensory clinical examination, all non-diabetic patients signed an informed consent form. Sensory testing, follow-up, and data collection were performed on Day 1, Day 10, and Day 14 of the infection and recovery periods (S1 Data). The selected testing days were based on a return-to-work permit suggestion for Day 10 and a recovery acceptance date of Day 14, which are timeframes that are consistent with those of most countries, including Saudi Arabia, where the research was conducted. The study was performed between September 19^th^, 2021 and July 20^th^, 2022, at King Fahad General Hospital (KFGH) in Jeddah, Saudi Arabia, which served as a COVID-19 center during that period. The demographic data was manually extracted from the electronic health record of KFGH on Excel sheet. The study was approved by the Research and Studies Department of Jeddah Health Affairs and the Ministry of Health and was registered with the King Abdulaziz City for Science and Technology (KACST) under research numbers H-020J-002 and IRB #1529. The research followed the Code of Ethics of the World Medical Association (Declaration of Helsinki).

### Sample and sensory testing procedure

The present study aimed to determine the sweet detection thresholds and variations among samples based on a sweetness index. Sugar solutions were prepared and tested using a pair-wise comparison procedure and sample concentrations were selected according to mean sweetness thresholds described in the literature [1]. Log differences of 0.25 were used between each concentration level, as listed in Table 1 [1, 32]. The solutions were then arranged in ascending order according to the just noticeable difference (JND) test protocol, dispensed into transparent plastic cups (25 ml) with a concealed 3-digit blinded code, and placed under daylight conditions. The JND test was used to determine the minimum sucrose concentration difference (threshold) that a participant could detect. The sweetness threshold of each participant was determined by presenting samples for tasting in ascending order of sucrose concentration and noting the lowest detected concentration after three consecutive positive detections. The median JND value was then calculated to represent the population average threshold based on the cumulative population tabulated against logarithmic stimulus levels.

**Table 1:** Summary of used sucrose solutions with varying concentrations presented in molarity and weight/volume (%). It includes the sweetness detection threshold of healthy individuals which was determined to be 0.23% sucrose concentration in Sample 4.

Sample No.	Molar concentration %	Sucrose concentration % (weight/volume)
**1**	0.0012	0.04
**2**	0.0021	0.07
**3**	0.0038	0.13
**4**	0.0068	0.23
**5**	0.0121	0.41
**6**	0.0215	0.74
**7**	0.0384	1.31
**8**	0.0683	2.34
**9**	0.1215	4.16
**10**	0.2163	7.40
**11**	0.3850	13.18
**12**	0.6853	23.46
**13**	1.2198	41.75

### Statistical analysis

This study is classified as a pilot study because similar study designs were not identified in the literature. Findings of the sensory tests were calculated using a probit analysis to observe the lognormal best fits, with confidence intervals calculated using Microsoft Office Excel 2016 (v16.0). The statistical analysis was conducted with XLSTAT (Microsoft, Mountain View, CA, USA) and Microsoft Office Excel 2016 (v16.0).

## Results

Thirty-seven volunteer patients aged 18 to 53 years (36.05±10.4), consisting of 18 women and 19 men, were deemed eligible to participate in the study based on the inclusion criteria. The demographics of the COVID-19 patients are presented in Table 2. A high incidence of loss of taste was reported in the patients on the first day (78.4%), which decreased over time to a proportion of 37.8 and 2.7% on Days 10 and 14, respectively. In contrast, the loss of smell showed a lower incidence with a progressive decrease over the testing period of 16.2, 13.5, and 5.4%, respectively for the same testing days. Thus, the study focused on the sensation of taste over that of smell, with a particular emphasis on sweetness thresholds.

**Table 2:** Detailed representation of demographic data for COVID-19 patients (n = 37 patients).

Factor		n (%)
**Gender**		
Male		19 (51.4)
Female		18 (48.6)
**Age**		
Young adult patients (18–25 years)		8 (48.6)
Adult patients (26–44 years)		18 (45.9)
Middle-aged patients (45–59 years)		11 (5.4)
Total age (Mean value ± SD)		36.05 ± 10.04
**BMI class**		
Normal weight (18.5–24.9)		23 (62.2)
Overweight (<24.9)		14 (37.8)
Total BMI (Mean value ± SD)		23.08 ± 2.93
**Smoking status**		
Smokers		19 (51.4)
Non-smoker		18 (48.6)
**COVID-19 symptoms ‐ Duration**		
Loss of taste	Day 1 Day 10 Day 14	29 (78.4) 14 (37.8) 1 (2.7)
Loss of smell	Day 1 Day 10 Day 14	6 (16.2) 5 (13.5) 2 (5.4)

## Discussion

Recent studies have indicated that sweet detection thresholds may differ between healthy individuals and cancer, diabetes, and obesity patients [33–37]. Healthy individuals generally have significant variations in sweetness detection abilities [1, 38], whereas patients with certain medical conditions such as cancers, diabetes, and obesity have shown alterations in their sweetness thresholds [33–37]. In addition, temporary losses or impairments of sweetness-tasting ability have been reported in COVID-19 patients [29–31, 39]. Therefore, determining sweet detection threshold alterations in COVID-19 patients could have significant implications for nutritional and dietary management.

**Fig 1:**
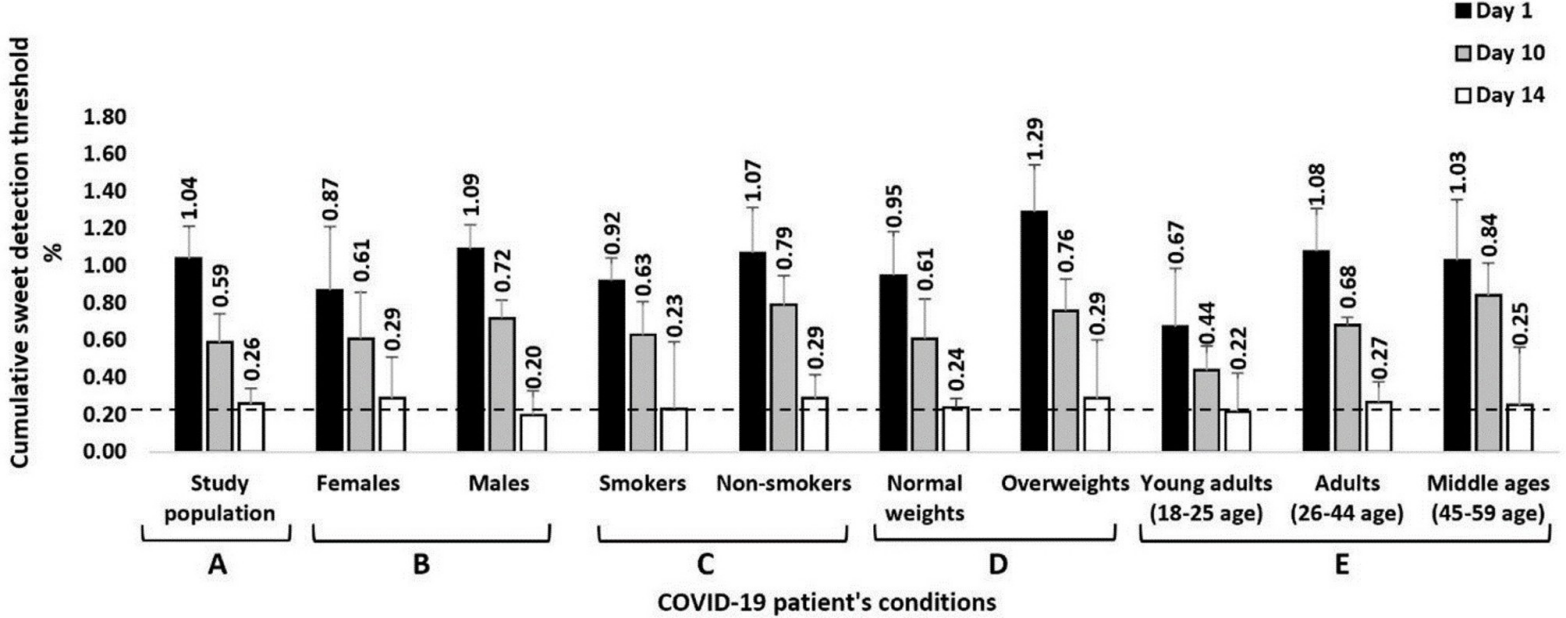
Summary of the cumulative sweet detection threshold results across different groups over the entire period of COVID-19 infection and recovery with taste loss or impairment (n = 37 patients). It is divided into three points: Day 1, Day 10, and Day 14, with a cross dashed-line representing the sweet detection threshold for healthy individuals at 0.23%. (A) study population, (B) gender, (C) smoking status, (D) BMI, (E) age.

The cumulative sweet detection threshold results in our study were divided into multiple groups based on the study population, gender, smoking status, BMI, and age (Fig 1). The results revealed a significant variance in the sweet detection thresholds of COVID-19 patients during infection onset and recovery (Fig 1A). A significant decrease in sweet detection thresholds occurred over the testing days, which indicated a consistent increase in sucrose sensitivity in the COVID-19 patients (higher sensation thresholds, suggesting lower sucrose sensitivities, on Day 1 compared to Days 10 and 14). Similar results were obtained by [40], whereby an increase in the sweet taste threshold was observed among COVID-19 patients and a marked reduction in sensation thresholds was evident during the recovery period. In addition, similar studies have reported that most patients recovered their sweet taste sensation within 14 days of infection with COVID-19 [29–31].

Several studies have shown that sweet taste sensation thresholds differ by gender [41–43]. When comparing the findings from this study on COVID-19 patients to those of a non-COVID-19 study on sweet detection thresholds, a similar tendency was observed regarding gender. Fig 1B demonstrates that on Day 1 of COVID-19 infection, men exhibited significantly higher sweet detection thresholds compared to infected women, which suggests a decreased sensitivity to sucrose in men compared to women. A non-COVID-19 study found similar results in terms of gender sensitivity to sucrose [44]. Men and women recovered within the same timeframe (Day 10), with their sweet detection thresholds showing comparable values (0.72 and 0.61%, respectively), suggesting that COVID-19 infection and recovery altered sweet detection similarly in both genders. Numerous studies have reported a rapid taste recovery in most patients [22, 25, 45, 46]. However, a gender-dependent association has been identified in the recovery of sweet detection thresholds following COVID-19 infection. Specifically, infected men demonstrated significant returns to their sweet detection thresholds on Day 14 of infection, which was not detected in infected women. Similar results were obtained by [44] for uninfected men. However, the literature reports inconsistent associations between sweet detection thresholds and gender. For example, some studies with non-COVID-19 patients have shown that women exhibit an increased taste acuity compared to men [41, 47], whereas others found no major difference in the response to sweet taste by gender [48]. The observed difference in sweet detection thresholds between infected men and women during the COVID-19 testing days may be related to differences in sex hormone levels (testosterone and estradiol for men and women, respectively) and their function, as previous research has shown that sex hormones can modulate immune responses [49–51]. These findings suggest that gender-related differences in sweet detection thresholds may be present in both non-COVID-19 individuals and COVID-19 patients.

A comparison of smoking and non-smoking COVID-19 patients during infection and recovery was performed to determine whether smoking influences sweet detection thresholds. In Fig 1C, the results suggested that smoking may impact sweet detection thresholds in COVID-19 patients during infection and recovery since smokers had lower sweet detection thresholds compared to non-smokers on Days 1 and 10 (more sensitive to low sucrose concentrations), whereas the results were similar between the groups on Day 14. The correlation coefficients showed that there was a negative relationship between smoking and the ability to detect sweet thresholds on all three testing days. Infected smokers exhibited greater recovery rates for sweet detection thresholds compared to infected non-smokers during the testing days. This conflicts with an earlier finding on non-COVID-19 smokers reported by [48], which indicated that smoking status had no impact on sweet taste. However, our result is consistent with that of [39], who suggested that a temporary abstinence from smoking during COVID-19 or the severity of COVID-19 infection symptoms could have influenced the differences in sweet detection thresholds between smokers and non-smokers. These findings highlight the potential benefit of smoking abstinence in enhancing taste recovery among COVID-19 patients.

Body mass index (BMI) is an influential factor that can affect sweet taste perception for both non-COVID-19 individuals and COVID-19 patients [43, 52]. The BMI results of this study identified significant differences in the sweet detection thresholds of COVID-19 patients based on weight group. Fig 1D shows that overweight patients appeared to have higher thresholds than normal-weight patients on Days 1 and 10. This result is supported by those of several other studies that found that non-COVID-19 populations with reduced sensitivities to sucrose levels had elevated sweet detection thresholds [16, 53, 54]. This increased sweet detection threshold could result from an increased consumption of sweet foods, which are often high in calories. This, in turn, could lead to obesity [54–57]. However, overweight patients tend to recover their sensitivity to sweet taste relatively quickly, reaching levels comparable to those of normal-weight patients by Day 14. A recent study conducted by [58] demonstrated this correlation between the duration of taste loss in COVID-19 overweight patients compared to that of normal-weight patients. This suggests that the weight status of COVID-19 patients may not have an enduring impact on sweet taste sensitivity. As patients recover from COVID-19, adopting healthy dietary habits could play a critical role in restoring their normal sensitivity to sweet taste, even among those who are overweight.

The study results identified a potential impact of age on the sweet detection threshold of COVID-19 patients. As shown in Fig 1E, young adults (aged 18–25) exhibited significantly lower detection thresholds for sweet taste, with values of 0.673, 0.439, and 0.218% for Days 1, 10, and 14, respectively. In contrast, adult patients (aged 26–44) and middle-aged patients (aged 45–59) had higher detection thresholds on all three testing days. On Day 14, adult and middle-aged patients appeared to experience recoveries of their sweet detection thresholds that were comparable to those of the younger group. These findings align with previous studies on sucrose thresholds in non-COVID-19 patients in which no decline was evident in the sweet taste sensation thresholds in association with age [44, 59]. The cross-dashed line in Fig 1 indicates a significant recovery of sweet detection thresholds across different COVID-19 patient groups on Day 14 when compared to a healthy individual with a threshold of 0.23%. Considering the relationships among different age groups and sweet detection thresholds in COVID-19 patients, these findings may be useful for developing dietary recommendations and interventions.

## Limitations

This pilot study is limited by its small sample size and lack of data on vaccine type, medical conditions, biomarkers, and dietary habits, which provides a rich opportunity for future research to explore these variables and increase the scope of findings, ultimately contributing to a more understanding of COVID-19’s effects on taste impairment.

## Conclusion

This study aimed to investigate the detection thresholds of sweet taste loss in confirmed COVID-19 patients, as well as the changes in the tasting abilities of these patients over time and across various demographics based on a sweetness index. The results revealed significant variances in the sweet detection thresholds of COVID-19 patients during the infection onset and recovery periods. The infected patients experienced consistently increased sucrose sensitivity over the testing days, with significant decreases in sweet detection thresholds. Gender, smoking status, BMI, and age group were identified as influential factors in determining the sweet detection threshold. Infected men exhibited significant recoveries of sweet detection thresholds on Day 14 compared to women, and significant differences were observed in the thresholds between smokers and non-smokers. Overweight patients had higher thresholds than normal-weight patients, and younger patients had lower sensory thresholds than older individuals for sweet taste. On Day 14, the sweet detection thresholds of the COVID-19 patients significantly recovered and reached a similar level to that of healthy individuals, who show values of approximately 0.23%. These findings highlight the importance of recognizing and managing alterations in sweet detection thresholds promptly in COVID-19 patients, as this could positively impact dietary management, nutritional recommendations, and interventions during the infection and recovery periods.

## Supporting information

S1 Data (XLSX)
